# The MHC-II transactivator CIITA, a viral restriction factor inhibiting the replication of Human T-Cell Lymphotropic Virus Type 1

**DOI:** 10.1186/1742-4690-8-S1-A171

**Published:** 2011-06-06

**Authors:** Giovanna Tosi, Greta Forlani, Vibeke Andresen, Marco Turci, Umberto Bertazzoni, Genoveffa Franchini, Roberto S Accolla

**Affiliations:** 1 Department of Experimental Medicine, University of Insubria, Varese, 21100, Italy; 2 Institute of Medicine, Hematology section, University of Bergen, Bergen, Norway; 3 Department of Life and Reproduction Sciences, Section of Biology and Genetics, University of Verona, Verona, Italy; 4 Animal Models and Retroviral Vaccines Section, National Cancer Institute, Bethesda, MD, USA

## 

Human T-cell Lymphotropic Virus type-1 (HTLV-1) is the causative agent of an aggressive malignancy of CD4+ T lymphocytes. It is believed that the viral transactivator Tax-1 is a major player in T-cell transformation. Thus, targeting Tax-1 protein is regarded as a possible strategy to arrest viral replication and ultimately to counteract neoplastic transformation.

Here, we demonstrated that CIITA, the master regulator of MHC class II gene transcription, inhibits HTLV-1 replication by blocking the transactivating function of Tax-1. Co-immunoprecipitation experiments have shown that CIITA and Tax-1 physically interact in vivo and that the first 108 amino acids of Tax-1 were necessary for this binding. Two adjacent regions (1-252 and 253-410) of CIITA bound independently to Tax-1, but only region 1-252 mediated Tax-1 inhibition, in agreement with the fact that CIITA residues from positions 64-124 were required to block Tax-1 transactivation. CIITA inhibitory action on Tax-1 function correlated with the nuclear localization of CIITA and was independent of the transcription factor NF-YB, previously involved in CIITA-mediated inhibition of Tax-2 of HTLV-2, a virus with still elusive pathogenic action. Furthermore, CIITA severely impaired the physical and functional interaction of Tax-1 with the cellular co-activator PCAF, which is required for the optimal activation of HTLV-1 promoter. Accordingly, the over-expression of PCAF restored Tax-1-dependent transactivation of the viral LTR promoter inhibited by CIITA.

These findings strongly support our original observation that CIITA, beside increasing the antigen-presenting function for pathogen antigens, acts as an endogenous restriction factor against human retroviruses by blocking virus replication and spreading.

